# Supplementary material for: Neuropixels 1536 Channel Quad Base probe reveals brain-wide communication underlying flexible sensorimotor sequences
Source: bioRxiv. 2026 Jul 27:2026.07.23.740388. Preprint. [Version 1] doi: 10.64898/2026.07.23.740388 (PMC13483804; doi:10.64898/2026.07.23.740388)
Supplement: Supplementary file 1 [file NIHPP2026.07.23.740388v1-supplement-1.pdf]

bioRxiv preprint doi: https://doi.org/10.64898/2026.07.23.740388; this version posted July 27, 2026. The copyright holder for this preprint (which was not certified by peer review) is the author/funder, who has granted bioRxiv a license to display the preprint in perpetuity. It is made available under a CC-BY 4.0 International license.

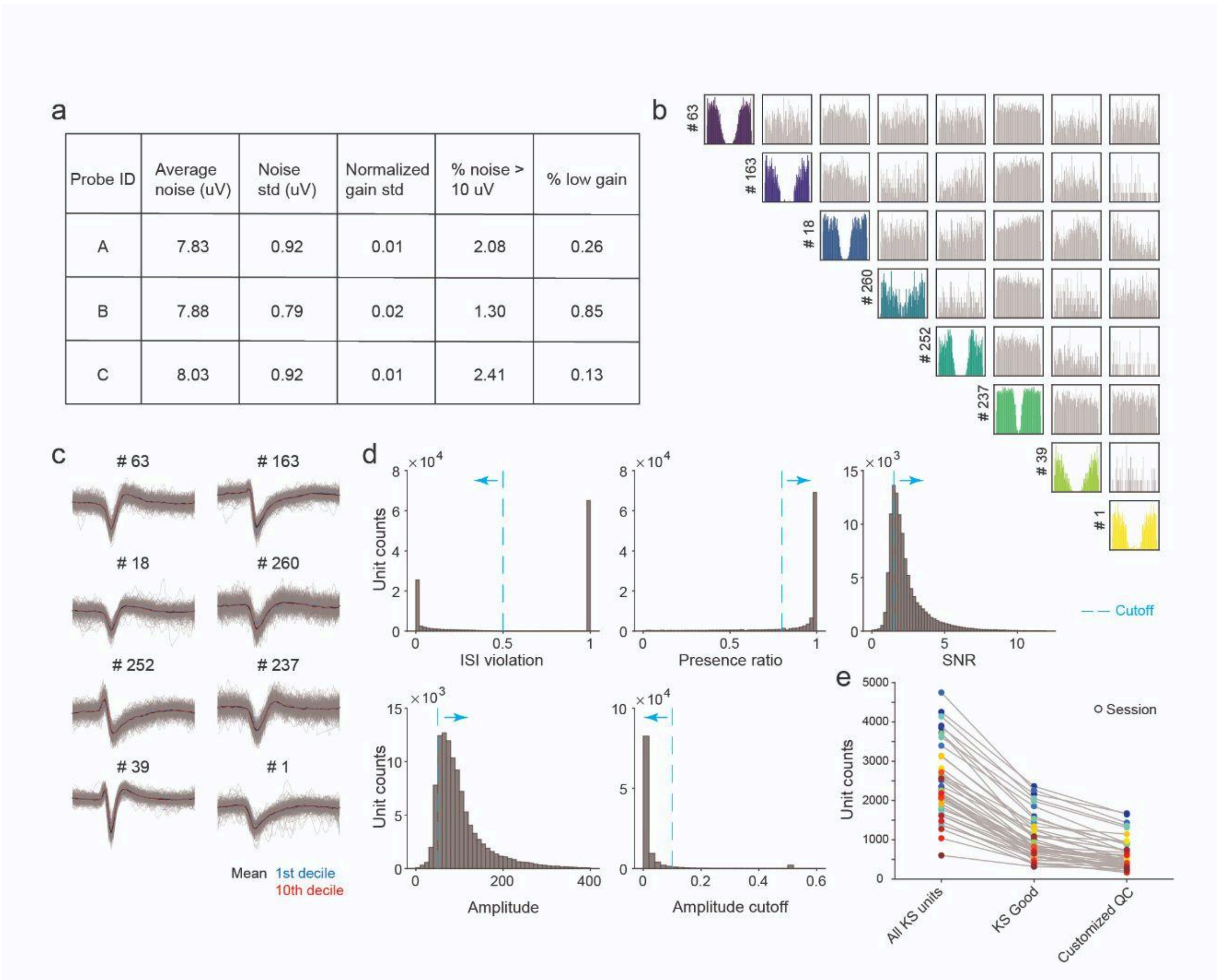

Probe ID	Average noise (uV)	Noise std (uV)	Normalized gain std	% noise > 10 uV	% low gain
A	7.83	0.92	0.01	2.08	0.26
B	7.88	0.79	0.02	1.30	0.85
C	8.03	0.92	0.01	2.41	0.13

Figure S1: Probe characterization, unit quality metrics, and selection criteria.

a, Statistical summary of noise and gain metrics in saline. "Low-gain" channels are defined as those with a gain value below 50% of the average; these channels were excluded from all other measurements.

b, Representative auto- and cross-correlograms of units in a recording session. Examples of autocorrelograms (ACGs) for isolated units and cross-correlograms (CCGs) between unit pairs following spike sorting. The clear refractory periods in the ACGs indicate high-quality single-unit isolation with minimal multi-unit contamination.

c, Stability of unit waveforms. Waveform profiles for the example units shown in (**b**). Each panel displays 300 randomly sampled spike waveforms (light grey) superimposed with the session mean (black). To demonstrate temporal stability, mean waveforms from the first decile (blue) and last decile (red) of the recording session are overlaid. The high degree of overlap between the early and late waveforms confirms the stability of the extracellular recordings and the consistency of unit isolation throughout the session.

bioRxiv preprint doi: https://doi.org/10.64898/2026.07.23.740388; this version posted July 27, 2026. The copyright holder for this preprint (which was not certified by peer review) is the author/funder, who has granted bioRxiv a license to display the preprint in perpetuity. It is made available under a CC-BY 4.0 International license.

d, Distribution of single-unit quality metrics. Histograms representing the distribution of metrics for all units identified by Kilosort. Cyan dashed lines indicate the customized thresholds applied to isolate high-quality single units. The criteria are: (1) inter-spike-interval (ISI) violations within a 1.5-ms window must be < 0.5 false positive rate; (2) spike presence must exceed 80% of the total session duration; (3) the signal-to-noise ratio (SNR) must be > 1.5; (4) mean unit amplitude must exceed 50 μV; and (5) the amplitude cutoff must be < 0.1 fraction of missing spikes.

e, Comparison of unit yields across quality control categories. The categories include: (1) all raw units identified by Kilosort; (2) units automatically labeled as "good" by the Kilosort algorithm; and (3) units meeting our customized quality control criteria (as defined in **d**). The use of the NP QB platform significantly maintains high unit yields even after the application of these stringent customized filters.

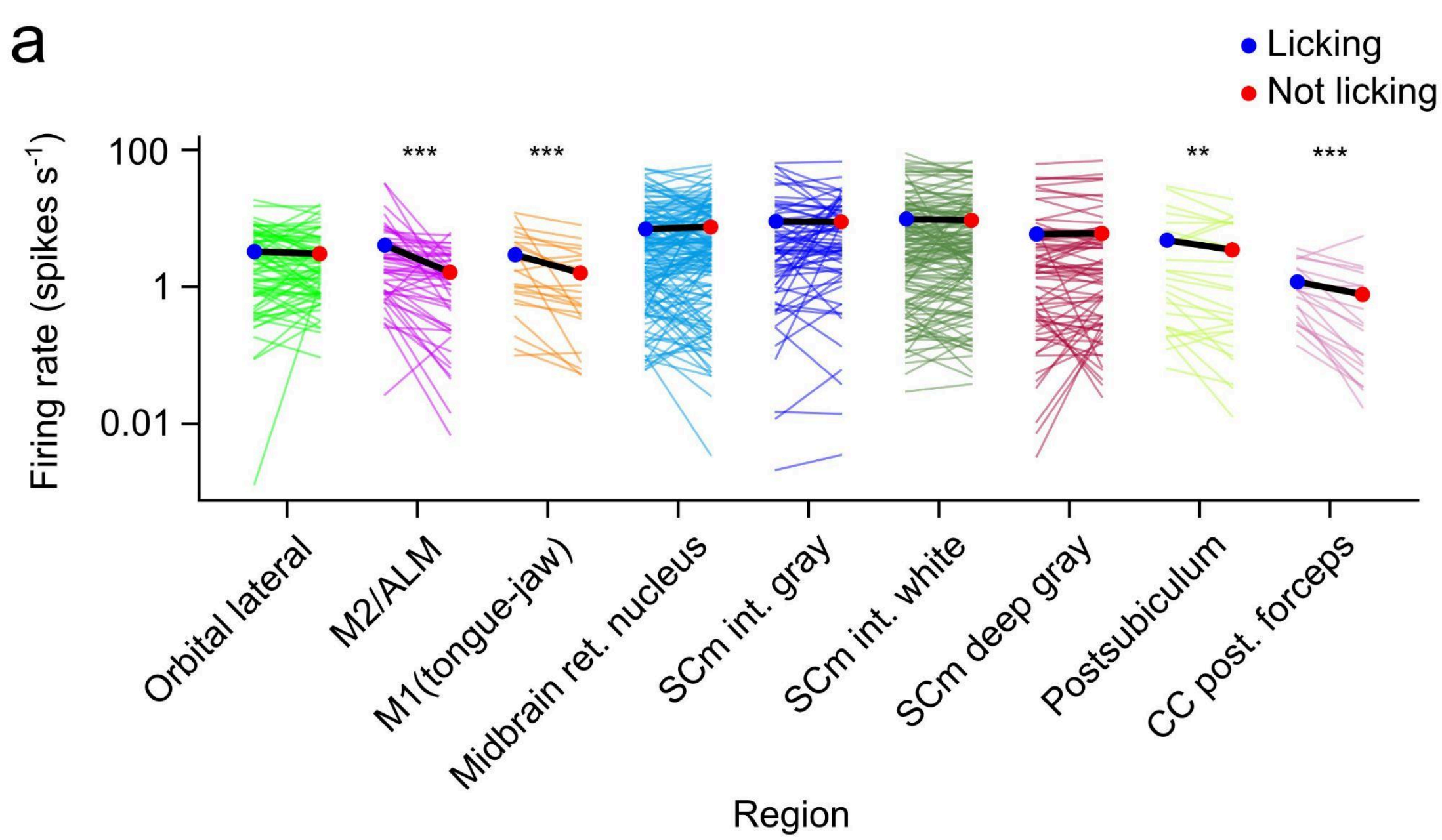

Figure S2: Firing rates of units shown in Figure 4 during Licking and Not licking periods.

a, Mean firing rates of units from different regions during the Licking and Not licking periods. Black lines show region-wise means. Two-sided Wilcoxon signed-rank test, *** $p < 0.001$, ** $p < 0.01$

bioRxiv preprint doi: https://doi.org/10.64898/2026.07.23.740388; this version posted July 27, 2026. The copyright holder for this preprint (which was not certified by peer review) is the author/funder, who has granted bioRxiv a license to display the preprint in perpetuity. It is made available under a CC-BY 4.0 International license.

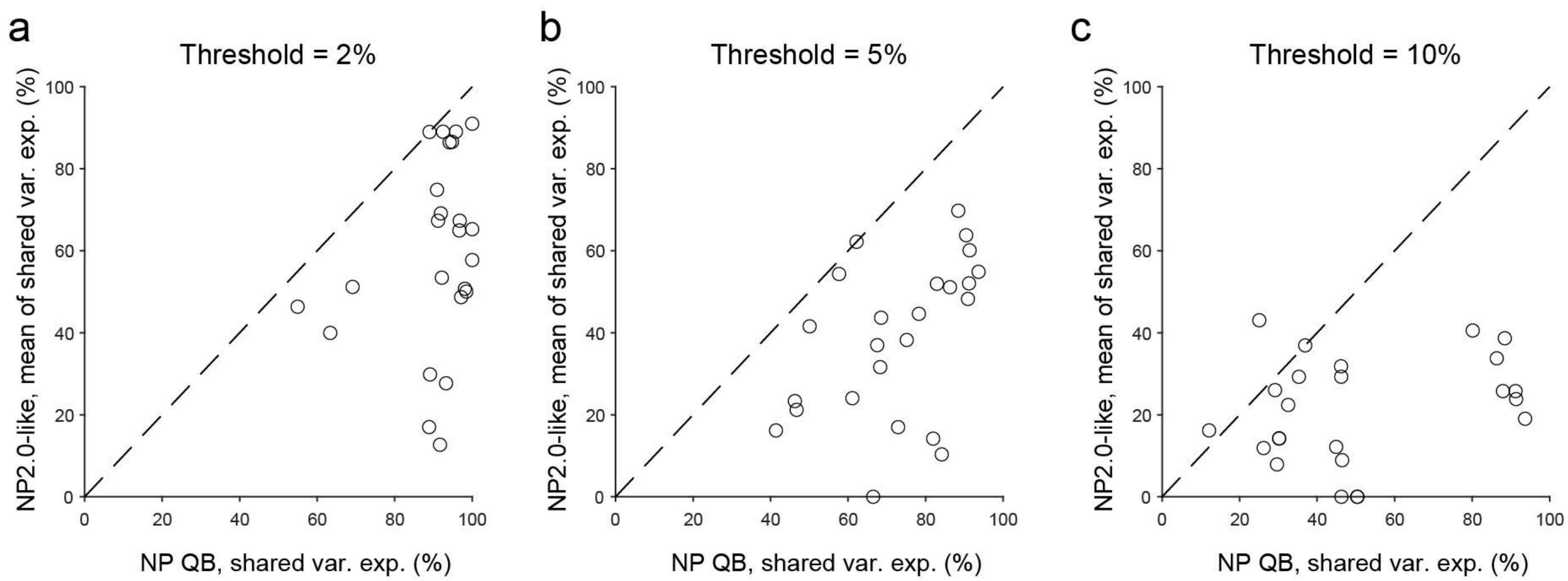

Figure S3: Shared variance explained of NP QB and NP2.0-like probes at different variance thresholds.

Shared variance explained from mDLAG analysis for NP QB probes is plotted against the mean of variance explained across the corresponding NP2.0-like simulations. Each plot shows analysis for a different variance threshold (2% in **a**, 5% in **b** and 10% in **c**).

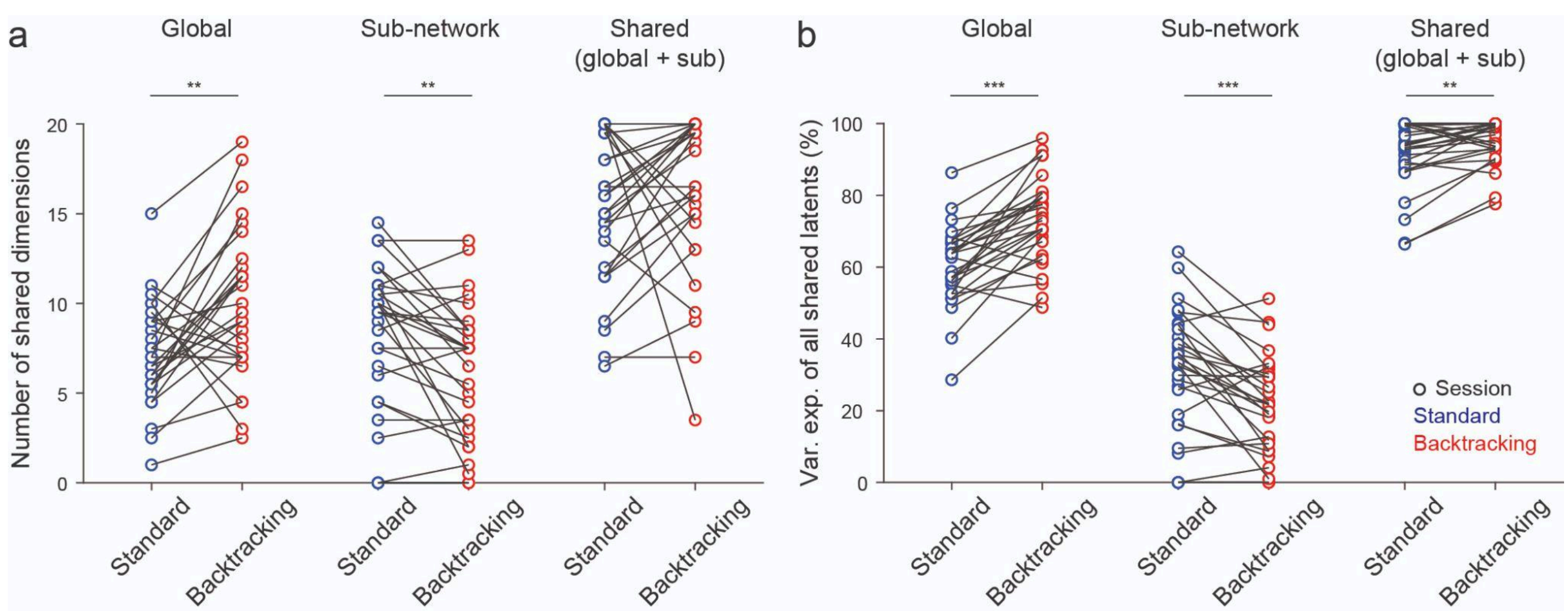

Figure S4: Comparison of latent dimensions and explained variance for standard and backtracking trials.

a, Number of latent dimensions identified with mDLAG analysis during standard (blue) and backtracking (red) trials. While the total number of shared dimensions (comprising both global and sub-network components) remains consistent across trial types, backtracking trials exhibit a significant shift in latent structure: global dimensions increase in number, while sub-network dimensions decrease.

bioRxiv preprint doi: https://doi.org/10.64898/2026.07.23.740388; this version posted July 27, 2026. The copyright holder for this preprint (which was not certified by peer review) is the author/funder, who has granted bioRxiv a license to display the preprint in perpetuity. It is made available under a CC-BY 4.0 International license.

b, Explained variance of latents in standard and backtracking trials. Total variance accounted for by all shared latent dimensions is significantly higher during backtracking trials compared to standard trials. This increased explanatory power is driven primarily by the global latent component.

Brain Region Name	Abbreviation
Agranular insular area dorsal part	Insular dors.
Agranular insular area ventral part	Insular vent.
Secondary motor area	M2/ALM
Primary motor area	M1(tongue-jaw)
Primary somatosensory area mouth	S1(tongue-jaw)
Primary somatosensory area nose	S1(nose)
Supplemental somatosensory area	S2
Primary visual area	V1
posteromedial visual area	PM visual
Substantia innominata	Subst. innom.
Pallidum	Pallidum
Globus pallidus external segment	GPe
Reticular nucleus of the thalamus	TRN
Anteromedial nucleus ventral part	AMV
Caudoputamen	CPu
Claustrum	Claustrum
Lateral dorsal nucleus of thalamus	LD thal.
Ventral posterolateral nucleus of the thalamus	VPL thal.
Anteroventral nucleus of thalamus	AV thal.
Ventral anterior-lateral complex of the thalamus	VAL thal.
Interanterodorsal nucleus of the thalamus	IAD thal.
external medullary lamina of the thalamus	EML thal.
Mediodorsal nucleus of thalamus	MD thal.
Lateral posterior nucleus of the thalamus	LP thal.
Posterior limiting nucleus of the thalamus	PL thal.
Thalamus	Thalamus
Orbital area lateral part	Orbital lateral
Orbital area medial part	Orbital medial
Orbital area ventrolateral part	Orbital ventrolat.
Midbrain reticular nucleus	Midbrain ret. nucleus
brachium of the superior colliculus	SC brachium
Superior colliculus motor related intermediate white layer	SCm int. white
Superior colliculus motor related deep white layer	SCm deep white
Superior colliculus motor related deep gray layer	SCm deep gray
Superior colliculus motor related intermediate gray layer	SCm int. gray
Superior colliculus superficial gray layer	SCs super. gray
Superior colliculus optic layer	SC optic
Superior colliculus zonal layer	SC zonal
Inferior colliculus central nucleus	IC central nucleus
Inferior colliculus dorsal nucleus	IC dorsal nucleus
Inferior colliculus external nucleus	IC external nucleus
internal capsule	Unknown
Anterodorsal nucleus	AD nucleus
Anteromedial nucleus dorsal part	AM nucleus dors.
Bed nuclei of the stria terminalis	Stria term. bed nucleus
stria terminalis	Stria term.
stria medullaris	Stria med.
third ventricle	3rd ventricle

bioRxiv preprint doi: https://doi.org/10.64898/2026.07.23.740388; this version posted July 27, 2026. The copyright holder for this preprint (which was not certified by peer review) is the author/funder, who has granted bioRxiv a license to display the preprint in perpetuity. It is made available under a CC-BY 4.0 International license.

fourth ventricle	4th ventricle
lateral ventricle	Lat. ventricle
corpus callosum anterior forceps	CC ant. forceps
corpus callosum posterior forceps	CC post. forceps
corpus callosum body	CC body
genu of corpus callosum	CC genu
Striatum	Striatum
fimbria	Fimbria
Septofimbrial nucleus	Septofimbr. nucleus
Retrosplenial area ventral part	Retrosplenial vent.
Retrosplenial area lateral agranular part	Retrosplenial lat.
Midbrain	Midbrain
cingulum bundle	Cingulum bundle
Subiculum	Subiculum
Postsubiculum	Postsubiculum
Prosubiculum	Prosubiculum
Hippocampal formation	Hippo. formation
hippocampal commissures	Hippo. commiss.
Field CA1	CA1
Dentate gyrus	Dentate gyrus
Periaqueductal gray	PAG
Cerebellum	Cerebellum
Pons	Pons
Anterior olfactory nucleus	Ant. olf. nucleus
Olfactory areas	Olfactory areas
Gustatory areas	Gustatory areas
Visceral area	Visceral area
fiber tracts	Fiber tracts
Endopiriform nucleus dorsal part	Endopiriform dors.
Anterior pretectal nucleus	Ant. pretect. nucleus
Cuneiform nucleus	Cuneiform nucleus
Parabrachial nucleus	Parabrach. nucleus
Pedunculopontine nucleus	Pedunculopont. nucleus
Medial vestibular nucleus	Medial vest. nucleus
Dorsal peduncular area	Dorsal pedunc. area
Fastigial nucleus	Fastigial nucleus
Posterior pretectal nucleus	Post. pretect. nucleus
Nucleus of the optic tract	Optic tract nucleus
supra-callosal cerebral white matter	Supra-callosal WM'
Frontal pole cerebral cortex	Frontal pole
Lateral septal nucleus rostral (rostroventral) part	Lat. sept. nucl. rostr.
Lobule II	Lobule II
Lobule III	Lobule III
Nucleus sagulum	Nucleus sagulum
Piriform area	Piriform area
Prelimbic area	Prelimbic area
Vestibulocerebellar nucleus	Vestibulocereb. nucleus
alveus	Alveus
arbor vitae	Arbor vitae
choroid plexus	Choroid plexus
cochlear nerve	Cochlear nerve
optic radiation	Optic radiation
uncinate fascicle	Unc. fascicle

bioRxiv preprint doi: https://doi.org/10.64898/2026.07.23.740388; this version posted July 27, 2026. The copyright holder for this preprint (which was not certified by peer review) is the author/funder, who has granted bioRxiv a license to display the preprint in perpetuity. It is made available under a CC-BY 4.0 International license.

Table S1: Full brain region names and corresponding abbreviations.

Probe tracks were reconstructed using SHARP-Track, and histological slice images were registered to the Allen Mouse Brain Common Coordinate Framework (CCF). This table maps the brain region names from the CCF to the corresponding abbreviations used in this study.
